# Wire arc directed energy deposition of AZ61 magnesium alloy fabricated using cold metal transfer

**DOI:** 10.1038/s41598-025-33668-2

**Published:** 2026-01-10

**Authors:** Jakub Slavíček, Čeněk Šváb, Stefan Gneiger, Jakub Hurník, Petr Procházka, Daniel Koutný

**Affiliations:** 1 Faculty of Mechanical Engineering, Institute of Machine and Industrial Design, Brno University of Technology, Brno, Czech Republic; 2 LKR Leichtmetallkompetenzzentrum Ranshofen, AIT Austrian Institute of Technology, Vienna, Austria; 3 Faculty of Electrical Engineering and Communication, Department of Power Electrical and Electronic engineering, Brno University of Technology, Brno, Czech Republic

**Keywords:** Magnesium alloy, Processing, Mechanical properties, CMT, WADED, WAAM, AZ61, Mechanical engineering, Metals and alloys

## Abstract

This research investigates the challenges and potential of Cold Metal Transfer (CMT) for Wire-Arc Additive Manufacturing (WAAM, also known as WADED – Wire-Arc Directed Energy Deposition) of AZ61 magnesium alloys. Despite the excellent properties of magnesium alloys, their processing is challenging due to high vapour pressure, low boiling point, flammability, and difficulties in maintaining a stable welding process. This study examines the effects of key CMT parameters, including boost phase current and duration, burn phase current, electrode speed, and short-circuit (SC) phase current. The results demonstrate that boost phase current, cycle time, droplet surface tension, and material consumption significantly influence deposition size and shape. By optimising these parameters, a stable and efficient welding process was achieved, improving contact angle and ensuring sufficient penetration. The optimised conditions enabled the fabrication of a 50-layer thin-walled component, highlighting the study’s contribution to advancing CMT applications in WADED processes.

Additive technologies are becoming increasingly important for producing complex and weight-saving shapes, offering advantages such as design flexibility, material efficiency, and reduced time to market^1^. Among the various AM approaches, Wire-arc Directed Energy Deposition (WADED), also referred to as Wire Arc Additive Manufacturing (WAAM), has attracted significant attention due to its high deposition rates, low feedstock and equipment cost, and ability to fabricate large-scale structures^1,2^. These advantages make WADED particularly suitable for applications in transportation and aerospace sectors, where lightweight and sustainable manufacturing solutions are essential.

Magnesium alloys are of special interest in this context because of their low density, high specific strength, and good thermal and damping properties, making them among the most promising structural materials for the 21st century^3–5^. However, their processing remains challenging. Limited ductility at room temperature due to the hexagonal close-packed (HCP) crystal structure, together with a low melting and boiling point, makes stable welding of magnesium alloys difficult^4–6^. This dilemma is further complicated by the difficulty in managing stable and continuous welding processes due to the physical properties of magnesium, such as its low density, low melting, and boiling

point^7,8^. Conventional welding methods often result in defects such as porosity, hot cracking, and unstable arc behaviour, which limit their widespread industrial use.

WADED can be implemented using several arc welding processes including gas tungsten arc welding (GTAW), plasma arc welding (PAW), or gas metal arc welding (GMAW)^9^. GTAW welding in combination with light metals is used mainly because of its high welding arc stability and lower weld fume formation^10^. The disadvantage of this system in combination with WADED is the need for additional equipment for welding torch rotation and the associated limitation of welding torch orientation when printing complex structures. In addition, GTAW productivity is approximately

2–3 times lower than GMAW^10^. GMAW is widely used due to its high efficiency, excellent gap-bridging capability, and compensates for the loss of alloying elements during welding^11^. However, excessive heat input during GMAW causes issues such as overheated droplets, grain coarsening, oxidation and evaporation, thermal stresses, and hot cracking^12^. To overcome these drawbacks, the Cold Metal Transfer (CMT) variant of GMAW was developed, which retracts the wire during the short-circuit phase and thereby lowers the heat input and spatter^13^. CMT has shown promising results for the cladding and welding magnesium alloys by mitigating many issues like droplet explosion and thermal stress^14^.

Several recent studies have focused on CMT-WADED magnesium alloys processing^15,16^. Wang et al. investigated the microstructure and mechanical properties of AZ31 alloy samples produced using three different CMT and CMT-P (a combination of CMT and pulse welding) parameter settings^17^. Their results showed that welding characteristics and parameters such as *I_boost*,* t_I_boost*,* Isc_wait*,* vd_sc_wait*, and *I_sc_2* significantly affect the shape of the deposit. However, they only compared the outcomes of three parameter sets without analysing the influence of individual parameters in detail. Manjhi et al.^18^ and Zhang et al.^19^ studied the effect of travel speed (TS) and wire feed speed (WFS) in CMT-WADED processing of AZ31. They demonstrated that these parameters strongly influence deposit geometry, but their work did not address the role of specific CMT characteristics or other key parameters. Similarly, Bi et al.^20^ examined the microstructure and mechanical properties of WADED AZ91 alloy, focusing on producing thick-walled components using a triangular weaving mode. While their study provided valuable insights, it did not analyse the effect of individual parameters on deposit shape. Gneiget et al.^21^ explored WADED processing of AZ61 magnesium alloy using CMT, but this alloy was briefly discussed. Their study’s primary aim was to develop a new alloy (AEX11) and compare its microstructure and mechanical properties with AZ61, without a detailed analysis of process parameters. In contrast, Ying et al.^22^ investigated WADED of AZ61 alloy using a GTAW heat source, partially considering the effect of processing parameters. Additional studies have also examined GTAW-WADED of AZ31^23^ and AZ80M^24^ alloys. However, due to fundamental differences between GTAW and CMT processes, findings from GTAW cannot be directly applied to CMT. Therefore, a systematic investigation of individual CMT process parameters in WADED of magnesium alloys is still needed to clarify their role in controlling deposit geometry, microstructure, and mechanical properties.

Previous studies have demonstrated the potential of CMT for magnesium alloy processing and provided insights into the influence of selected parameters such as wire feed speed (WFS), travel speed (TS), different process modes (CMT, CMT-P, CMT-ADV), or deposition strategies (e.g., triangular weaving). However, a comprehensive description of the fundamental arc behaviour, specifically at the level of characteristic parameter adjustment, is still missing. In particular, the link between the current and voltage waveforms of the arc and the resulting deposit geometry has not yet been systematically established. Since deposit geometry directly affects build quality and process reliability, clarifying this correlation represents a critical step towards the effective industrial implementation of WADED for magnesium alloys.

This study provides a detailed analysis of how the main CMT characteristic parameters affect the geometry of single deposits. The objective is to identify parameter settings that yield a favourable contact angle and sufficient deposition depth across the cross-section. By clarifying the role of key parameters in welding behaviour, the work establishes guidelines for the reliable production of AZ61 alloy. Furthermore, the microstructure and mechanical properties of thin-walled parts fabricated with the optimised parameter set are evaluated. These results are compared with properties of materials produced by alternative manufacturing routes, thereby assessing the potential of CMT-WADED for the fabrication of thin-walled AZ61 components.

## Materials and methods

**Fig. 1:**
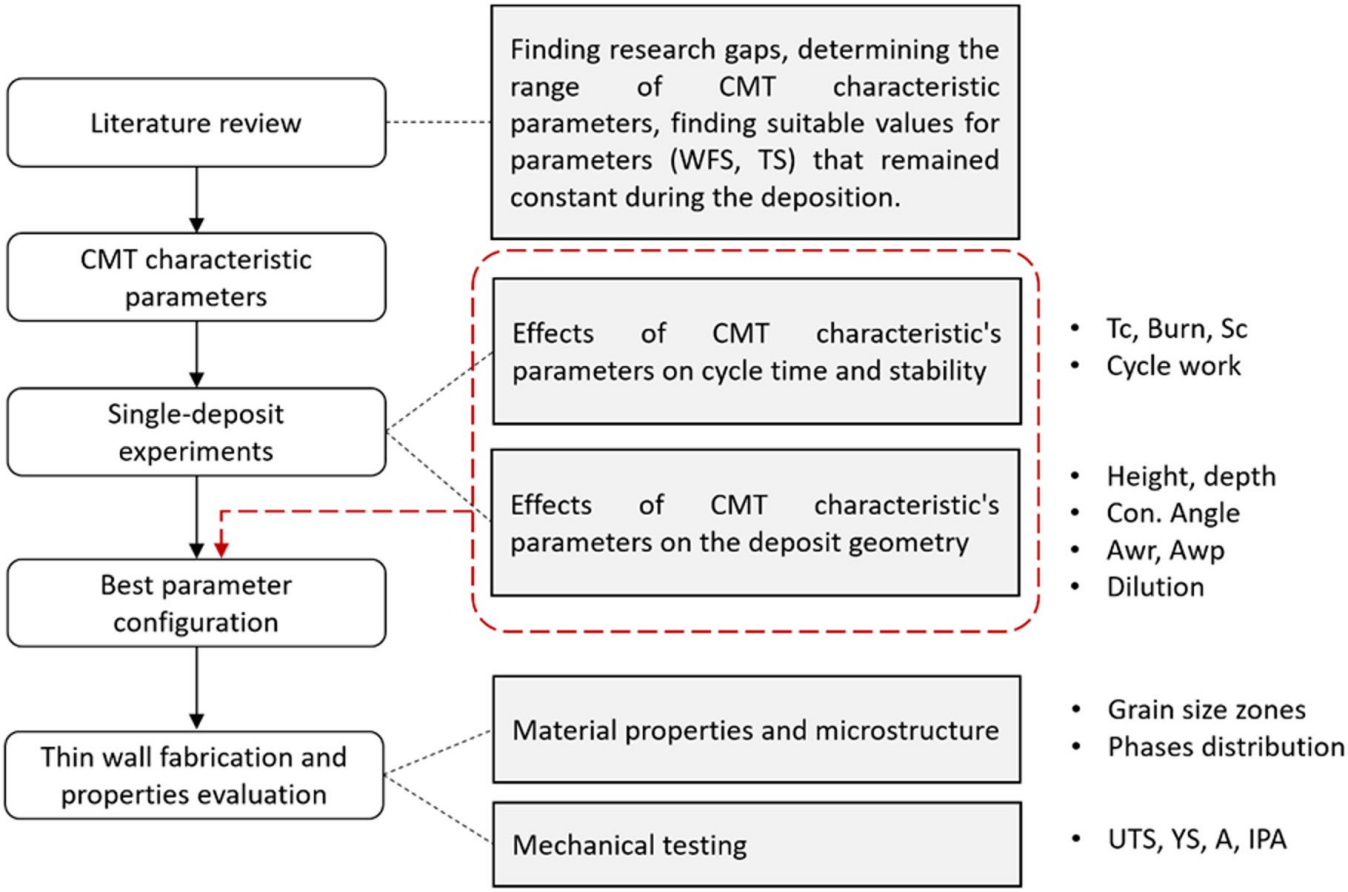
Flow chart of the research progress.

The overall research progress is summarised in Fig. 1. The study begins with a literature review to identify research gaps and relevant CMT parameters to establish a basis for comparison. Based on the review, the range of tested CMT characteristic parameters was determined and tested in single-deposit experiments to evaluate their influence on weld geometry and cycle stability. From these results, the most suitable parameter configuration was determined. The best parameter set was applied to thin-wall fabrication, followed by microstructural and mechanical testing. Each step provided outcomes that guided the next stage of the research.

### Process parameters

The single deposit test was conducted on the base material (BM) with 100 × 20 × 8 mm dimensions at room temperature before welding. The BM was fabricated from AZ91D magnesium alloy (composition in Table 1). AZ61 wire with a diameter of 1.6 mm was used as a filler material with a chemical composition provided by the distributor, as listed in Table 1. Deposits were made using a Fronius TPS 3200 CMT welding system (Fronius International GmbH, Pettenbach, Austria). The positioning movement of the welding torch was accomplished using a KUKA KR 60 HA robotic arm (KUKA AG, Augsburg, Germany).

**Table 1:** Chemical composition of AZ61 (wire) and AZ91 (BM) magnesium alloys.

Alloy	Elements	Al	Zn	Mn	Si	Fe	Cu	Ni
AZ61 (filler wire)	Min (%)	5.50	0.5	0.15				
	Max (%)	6.50	1.50	0.40	0.10	0.005	0.05	0.005
AZ91 (base material)	Min (%)	8.30	0.35					
	Max (%)	9.70	1.00	0.13	0.10	0.005	0.03	0.002

During the tests, a constant speed of the welding torch (WS) of 10 mm·s^− 1^ and a distance from the manufactured part of 13 mm was maintained. Pure argon (99.5%) was adopted as the shielding gas with a flow rate of 18 l·min^− 1^.

**Fig. 2:**
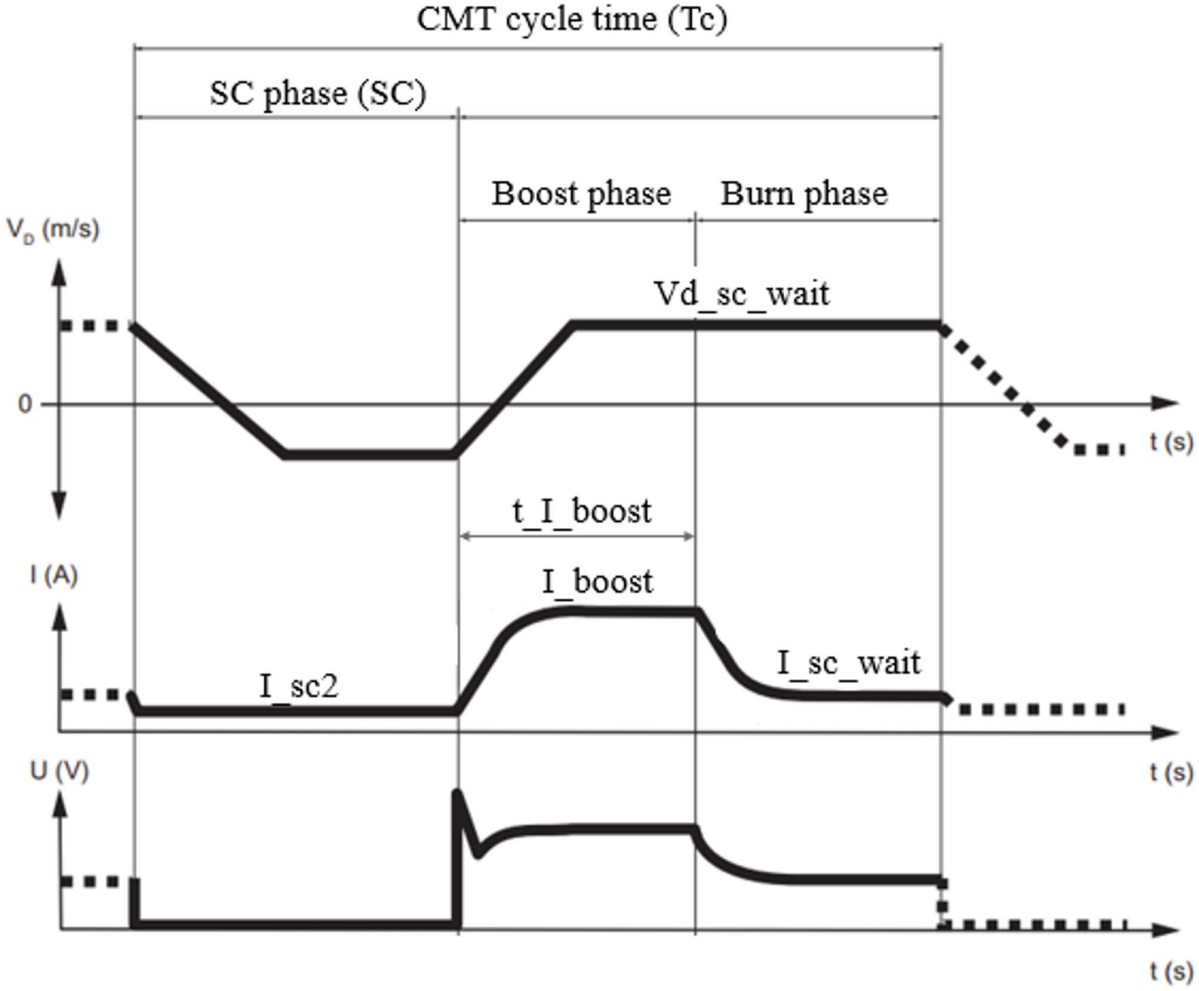
CMT characteristic welding process (Modified^25^).

A DC-CMT characteristic given by the synergic line (G3Sil), according to Wang et al.^17^, with modifications based on previous experiments was used as a mean value throughout the whole experiment. The characteristic is shown in Fig. 2 and consists of the 3 main phases: melting of the wire while the movement of the electrode goes upwards (boost phase), returning of the wire back to the weld pool (burn phase) and the contact and transfer of the melted droplet (SC phase).

The single-deposit experiment consisted of 21 combinations listed in Table 2 created by altering different parameters of the CMT characteristic. Five main characteristic parameters can be controlled during the CMT welding process. I_boost (A) - boost phase current, t_I_boost (ms) – boost phase duration, I_sc_wait (A) – burn phase current, vd_sc_wait (m·min^− 1^) – the speed of electrode during the boost and burn phase and I_sc2 (A) – SC phase current. The RCU 5000i controller was used to alter the synergic line and CMT parameters.

**Table 2:** The different settings of CMT characteristic used in experiment.

Deposit	I_boost	t_I_boost	I_sc_wait	vd_sc_vait	i_sc2
	(A)	(ms)	(A)	(m·min-1)	(A)
**1**	**360**	2	35	30	40
**2**	**400**	2	35	30	40
**3**	**430**	2	35	30	40
**4**	**450**	2	35	30	40
**5**	**480**	2	35	30	40
**6**	430	**1.7**	35	30	40
**7**	430	**2.3**	35	30	40
**8**	430	**2.6**	35	30	40
**9**	430	**3**	35	30	40
**10**	430	2	**15**	30	40
**11**	430	2	**55**	30	40
**12**	430	2	**75**	30	40
**13**	430	2	**100**	30	40
**14**	430	2	35	**10**	40
**15**	430	2	35	**20**	40
**16**	430	2	35	**45**	40
**17**	430	2	35	**60**	40
**18**	430	2	35	30	**20**
**19**	430	2	35	30	**60**
**20**	430	2	35	30	**80**
**21**	430	2	35	30	**100**

**Fig. 3:**
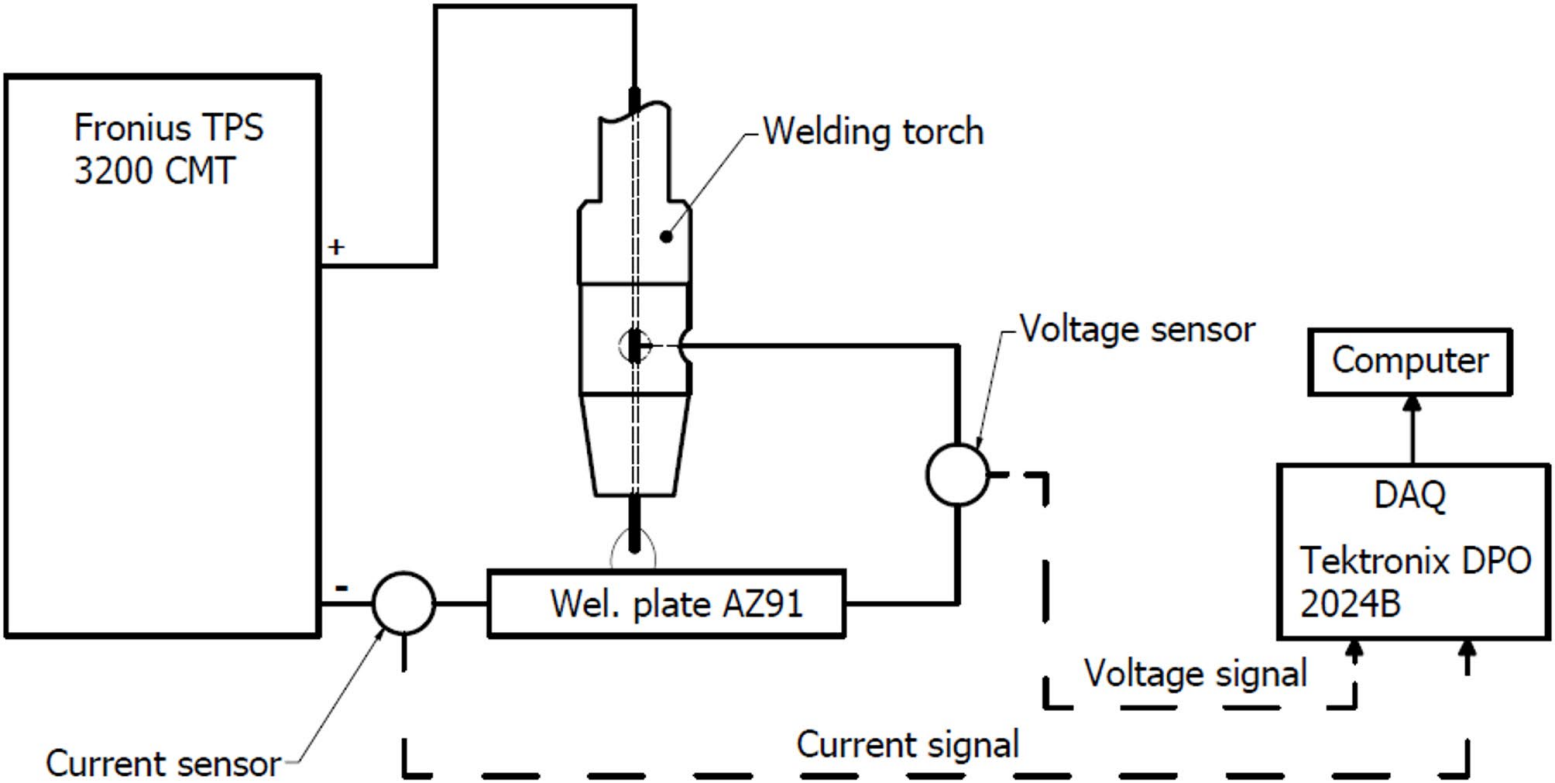
Schematic of the experimental set-up of CMT welding process.

For an accurate description of how individual parameters affect the geometry of a single deposit, it is essential to precisely measure the current and voltage signals of the welding arc. Figure 3 presents a schematic diagram of the experimental setup for recording the characteristic waveforms. The voltage and current signals were captured with a Tektronix DPO 2024B (Tektronix, Beaverton, OR, USA) oscilloscope at a sampling frequency of 312.5 kHz.

Individual phases of the CMT cycle were measured based on diagram given in Fig. 1. The average power of the cycle was calculated by the value of voltage ($\:{U}_{\left(x\right)}$) and current ($\:{I}_{\left(x\right)}$) in every step (n) by Eq. 1:

1 $$\:{P}_{c}=\frac{1}{n}\sum\:_{x=0}^{n}{U}_{\left(x\right)}\bullet\:{I}_{\left(x\right)}$$

### Geometry analysis, dimensions of samples

**Fig. 4:**
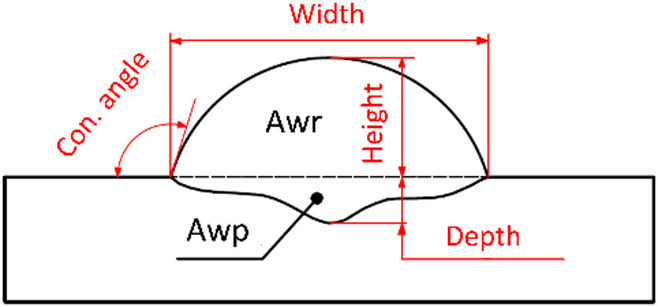
Schematic of geometry of deposition.

Overall, 21 samples were fabricated and analysed, individual samples were cut, and cross-sections were polished using a series of 3 grinding discs with grit sizes: P320, P500, and P1000. Subsequently, they were polished with a P3000 paste, and the geometry and macrostructure of the deposits were observed using an Olympus SZX7 microscope (OLYMPUS, Tokio, Japan). A diagram of the main dimensions of the deposition is shown in Fig. 4. Awp represents the penetration area (below the width line). Awr indicates the area of reinforcement (above the line).

**Fig. 5:**
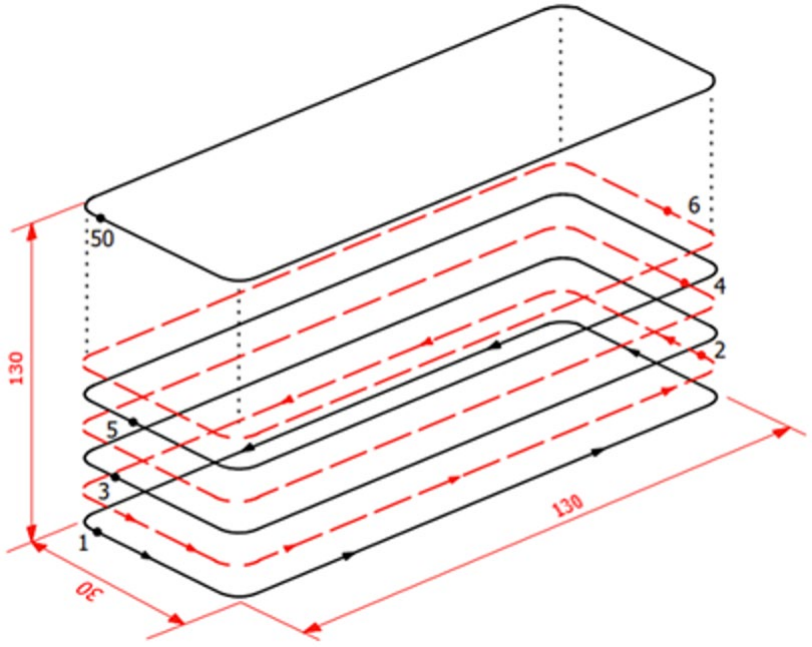
The schematic diagram of the planned tool paths for production of the thin-walled component.

Based on the findings obtained from the comprehensive parametric study, a set of optimized parameters suitable for WADED was determined. The schematic diagram of the CMT-WADED process is depicted in Fig. 5. The component dimensions were designed to allow the extraction of tensile specimens according to DIN 50,125. The start and end points of the trajectories were positioned outside the regions designated for specimen machining to avoid local inhomogeneities in the tensile samples.

A 100 s interlayer waiting time was applied. Using this methodology, a thin-walled component consisting of 50 layers with dimensions of 130 mm x 30 mm x 130 mm was successfully fabricated.

### Material properties and microstructure

Material properties were investigated to describe the achieved microstructure (defects, material phases, grain size) and clarify the material’s mechanical behaviour.

Thermodynamic calculations in the Equilibrium state and using the Scheil model were performed using Thermo-Calc software (Version 2024a) and database TCMG6 (Thermo-Calc Software AB, Solna, Sweden). The calculation was carried out using the average chemical composition in Table 1.

Microstructural investigations were done by means of optical light microscopy (OLM) and scanning electron microscopy (SEM) on polished and polished + etched (picric acid) samples. For OLM, an optical microscope OLYMPUS BX53M with a digital camera OLYMPUS SC180 (OLYMPUS, Tokio, Japan) was used, and the microstructural images were taken by the software OLYMPUS Stream Motion 2.5. A Tescan MIRA 3 scanning electron microscope (TESCAN, Brno, Czech Republic) equipped with a field emission gun (FEG) operated at 15 kV was used for SEM analysis. The electron images were taken using a four-quadrant backscattered electron detector (TESCAN, Brno, Czech Republic) operated at a working distance of 15 mm. Energy-dispersive X-ray spectroscopy (EDS) was performed using an Octane Elect silicon drift detector (EDAX, Mahwah, NJ, USA). The fracture surfaces of the tensile specimens were examined using a digital light microscope (Keyence VHX-6000, Z250R lens, 250× magnification).

### Mechanical testing

**Fig. 6:**
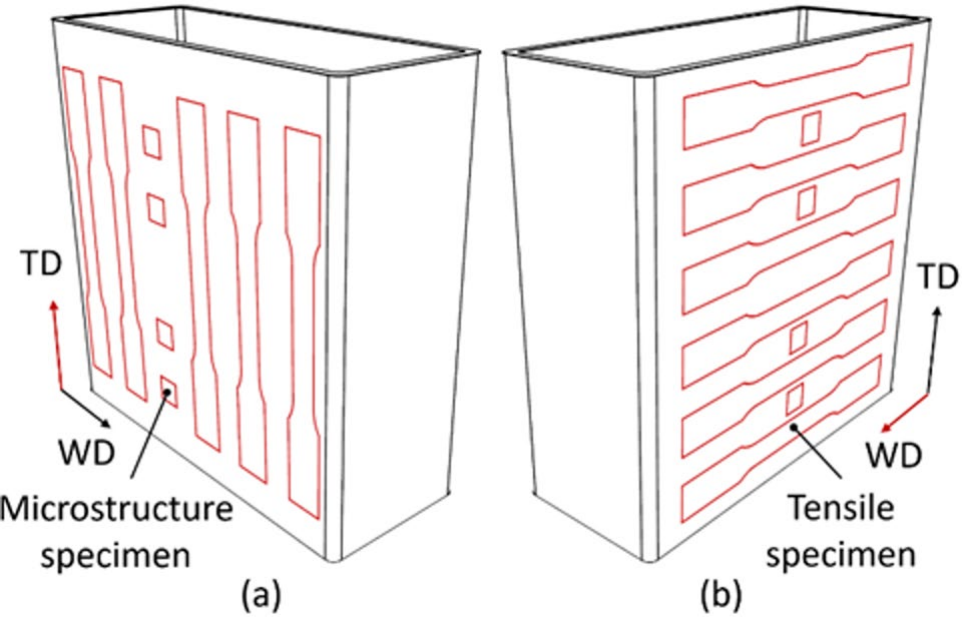
Schematic illustration of specimen extraction: (**a**) transversal direction (TD); (**b**) welding direction (WD).

The samples for microstructural investigation and tensile tests were extracted from the thin-walled part in the way you can see in picture Fig. 6. The tensile specimens had dimensions specified by DIN 50125 (type E) and a thickness of 3 mm. Tensile tests were conducted at room temperature using a universal testing machine (Shimadzu AGX-V 100kN, Shimadzu Corporation, Kyoto, Japan). The deformation rate during testing was 0.5 mm·min^− 1^.

Ductility measurements were performed using a stereo 3D digital image correlation (DIC) system (Dantec Dynamics, Skovlunde, Denmark). The system incorporated two 5 MPx cameras (2448 × 2048 px, 3.45 μm pixel size) with 50 mm lenses. A baseline of 160 mm provided a 17° stereo angle appropriate for planar samples. The frame rate was set at 0.5 Hz. The correlation parameters were adjusted for better reliability – a facet size of 29 pixels with a spacing of 19 pixels was used.

The in-plane anisotropy percentage (%IPA) can be calculated to assess the degree of anisotropy in the tensile properties. The %IPA(X), where $\:\mathrm{x}$ represents the tensile property index, was calculated by the maximum value obtained from the building and travel directions ($\:{X}_{max}$) and the minimum value from the building and travel directions ($\:{X}_{min}$) by Eq. 2^26^:

2 $$\:\mathrm{\%}\mathrm{I}\mathrm{P}\mathrm{A}\left(\mathrm{x}\right)=\frac{{X}_{max\:-}{X}_{min}}{{X}_{max}}$$

## Results and discussion

### Effect of CMT characteristich

#### Boost phase current (I_boost)

**Fig. 7:**
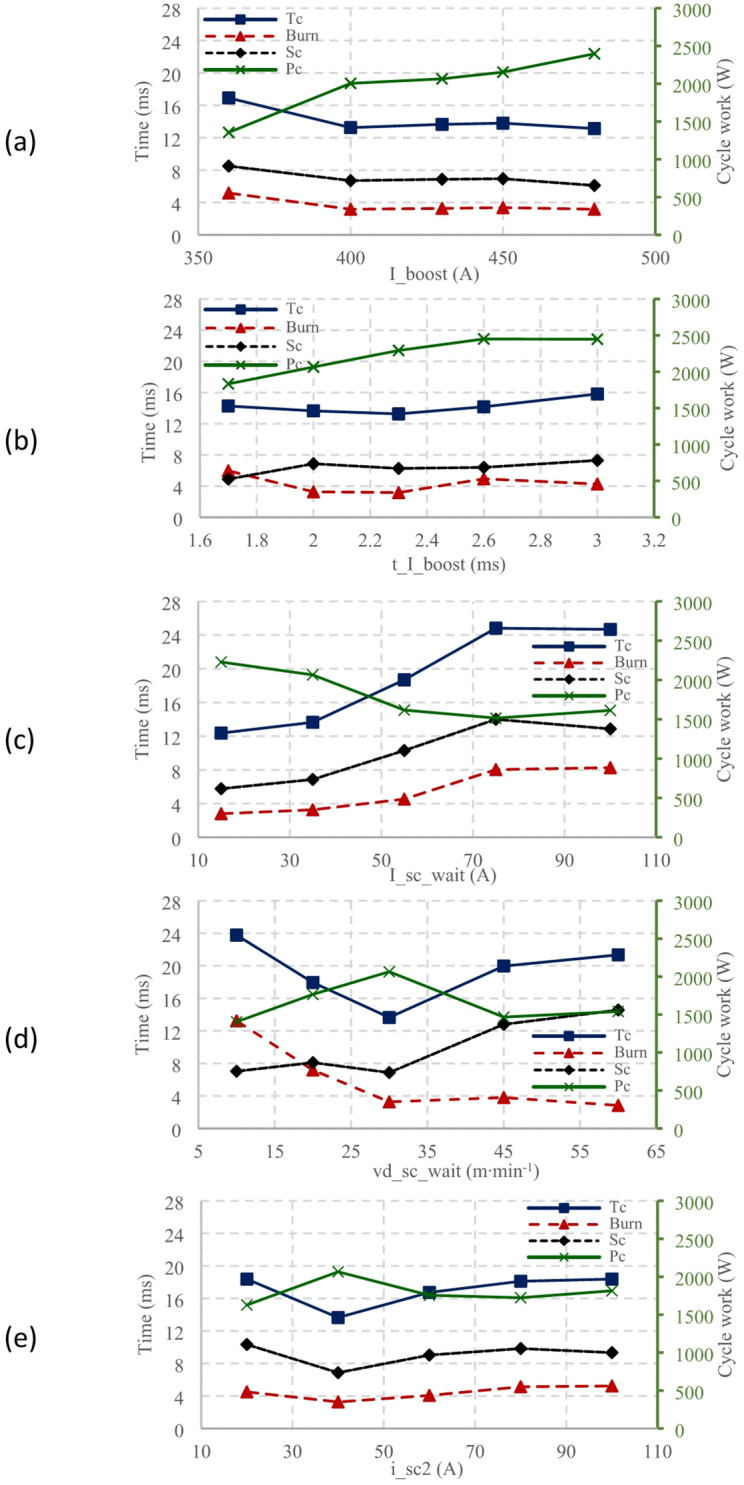
Influence of characteristic parameters on energy input of CMT welding process: (**a**) I_boost, (**b**) t_I_boost, (**c**) I_sv_wait, (**d**) vd_sc_wait, (**e**) I_sc2.

Increasing the current from 360 A to 480 A in the welding process led to noteworthy changes in heat input, deposition geometry, and cycle times. Initially, at 360 A, the heat input (Pc) was 1356.1 W, but it rose significantly to 2004.9 W at 400 A and further to 2396.7 W at the peak current of 480 A, see in Fig. 7 (a). Concurrently, the cycle time (Tc) was the longest (16.9 ms) at 360 A and then decreased sharply to 13.2 ms. With the further increase in current, the cycle times showed minor fluctuation.

The sudden drop in cycle time observed between 360 A and 400 A is atypical. As demonstrated in He’s study^27^, an increase in current typically leads to an increase in droplet size. Given that the droplet diameter exceeds the wire diameter, the duration of the burn phase would be expected to increase accordingly. However, as shown by^28^, an increase in droplet size does not necessarily result in a longer burn time; instead, the burn phase duration remains relatively consistent despite the rising boost current. A similar trend was observed in this study in Fig. 7 (a) while increasing the boost current from 400 A to 480 A.

**Fig. 8:**
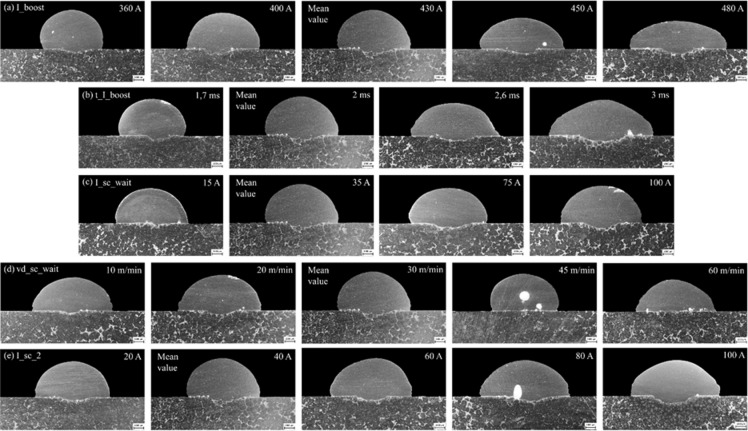
Influence of parameters on deposition geometry: (**a**) I_boost, (**b**) t_I_boost, (**c**) I_sc_wait, (**d**) vd_sc_wait, (**e**) I_sc2.

**Fig. 9:**
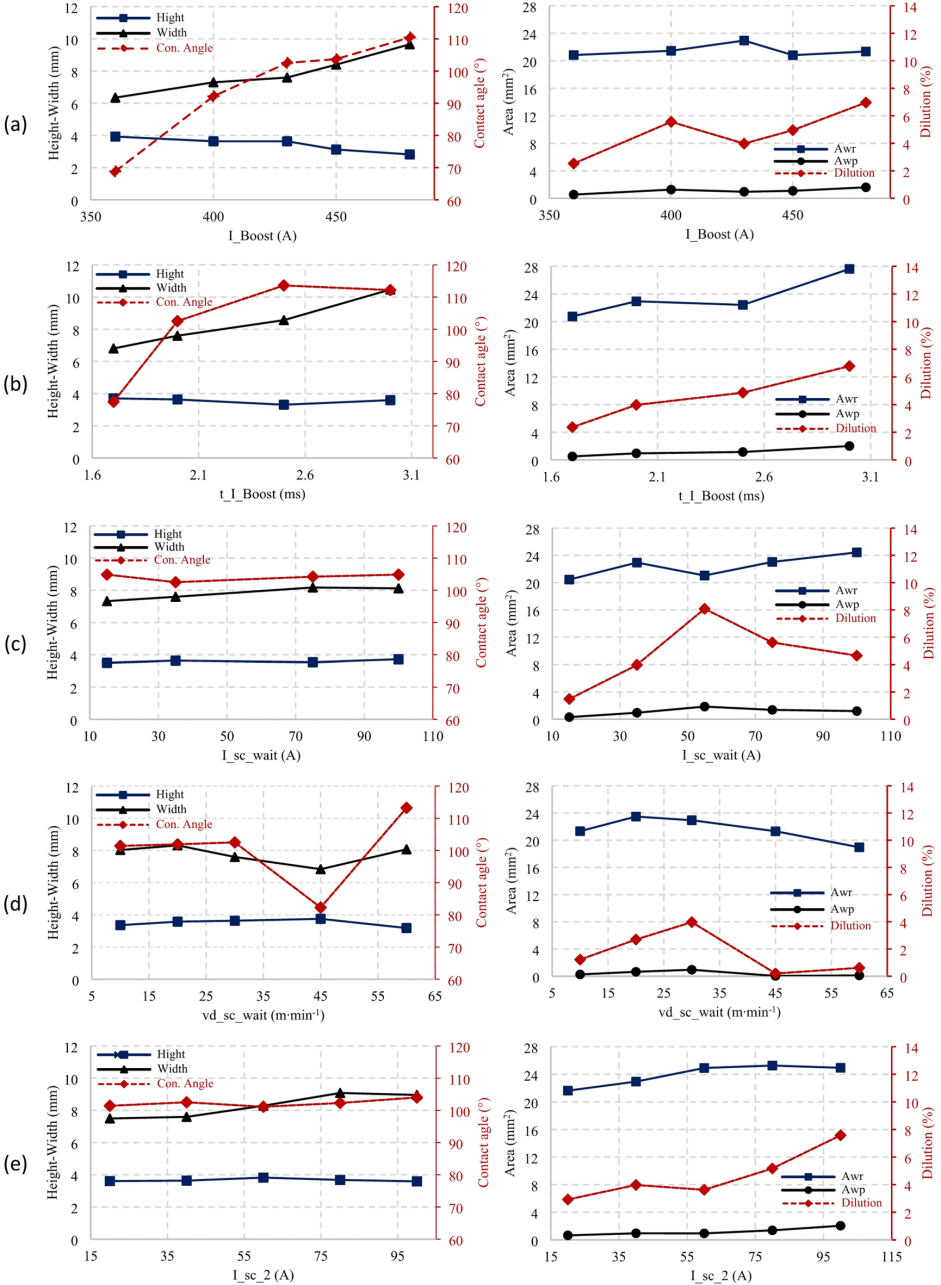
Influence of characteristic parameters on deposition geometry: (**a**) I_boost, (**b**) t_I_boost, (**c**) vd_sc_wait, (**e**) I_sc2, Awp – penetration area, Awr – reinforcement area.

Regarding deposition geometry, Fig. 8 (a) and Fig. 9 (a), an increase in current led to the width expansion from 6.3 mm to 9.6 mm and a decrease in its height from 3.9 mm to 2.8 mm. This change is attributed to the variable surface tension and a smaller weld pool size, leading to a greater spread of the droplet on contact with the weld pool^28^. The increase in current also resulted in an increased penetration depth from 0.34 mm to 0.51 mm. The table of precisely measured values of the deposit geometry, graphically represented in Fig. 8, is included in the dataset accompanying this publication.

The penetration area (Awp) showed some fluctuations. It increased from 0.53 mm^2^ (at 360 A) to 1.26 mm^2^ (at 400 A), followed by a minor reduction to 0.9 mm^2^ (at 430 A) and a final increase to 1.6 mm^2^ (at 480 A). The largest Awp area of 22.9 mm^2^ was achieved at 430 A with a wire feed speed (WFS) of 6.1 m·min^− 1^. However, the penetration area decreased slightly for currents of 450 A and 480 A. This was likely because the boost current exceeded the spray transition current, and the duration of the boost phase was long enough to allow for some spray transfer and accompanying spatter to occur^6,28^.

#### Boost phase duration (t_I_boost)

As the boost time increased, the average power also rose, reaching a peak of 2450 W at 3 ms. The total cycle time exhibited a convex trend, with a minimum duration of 13.2 ms observed at a boost time of 2.3 ms. The correlation of the trend in burn and short-circuit phases can be seen in the graph Fig. 7 (a) of the I_boost parameter. In contrast, in the graph Fig. 7 (b), increasing the t_I_boost did not lead to a similar obvious correlation between the mentioned parameters.

In deposition geometry Fig. 8 (b) and Fig. 9 (b), increasing the boost phase duration led to an increase in the deposition’s width (from 7.1 mm to 10.9 mm) and minimal decrease in its height (from 3.9 mm to 3.7 mm), which is well within the margin of error. These geometry changes resulted in a higher contact angle from 68° to 110° and an increased weld penetration depth from 0.34 mm to 0.73 mm.

Increased boost phase duration expanded the weld bead area. It led to no significant variation in bead height, but a noticeable increase in bead width. When the parameter was set to 3 ms, a substantial increase in Awr, followed by a slight increase in Awp, was observed. Simultaneously, a reduced cycle length followed the same pattern as Fig. 7 (a) at the beginning. This reduction was due to an extended burn phase at a boost phase duration of 1.7 ms, likely because the boost phase ended before the electrode’s oscillatory movement^28^. The overall cycle length increased for boost phase durations of 2.6 ms and 3 ms due to the larger droplet size.

#### Burn phase current (I_sc_wait)

The highest average cycle work of 2228.5 W was measured at a current of 15 A. Changes in this parameter showed a strong correlation with the current magnitude, akin to the I_boost and t_I_boost parameters, with exceptions observed at 75 A and 100 A due to increased cycle durations reaching 24.8 ms and 24.6 ms, respectively. These duration changes led to corresponding increases in both the burn and SC phases, as illustrated in Fig. 7 (c).

Only slight increases in height, width, and contact angle with the I_sc_wait parameter were seen, unlike the significant changes in the other parameters. However, weld penetration depth did increase from 0.27 mm at 15 A to 0.59 mm at 100 A, as per Fig. 8 (c) and Fig. 9 (c). The wire feed speed remained constant at 6 m·min^− 1^ for 35 A and 75 A.

The increase in the overall cycle time and the duration of each phase can be attributed to the interaction of two factors. During the boost phase, the droplet is melted, and upon transition to the burn phase, the continued current input supports further droplet growth. Simultaneously, the magnitude of the burn phase current induces a recoil effect, leading to droplet flattening and consequently prolonging the burn phase. When the droplet eventually contacts the weld pool, the electrode position is lower, and its subsequent emergence from the solidifying weld pool takes longer, thereby extending the SC phase. A similar recoil behaviour was observed in Gomez’s study^29^.

#### Electrode speed during the boost and burn phase (vd_sc_wait)

The study found the average cycle work mirrored the pattern of cycle length, peaking (2065 W) at the minimum cycle length of 13.6 ms and hitting a low (1404 W) at the maximum cycle length of 23.7 ms. In terms of geometry, the increase in speed leads to a slight increase in contact angle from 101° at 10 m·min^− 1^ to 115° at 60 m·min-1, except at 45 m·min^− 1^, where the contact angle dropped to 82°, as shown in Fig. 9 (d). Penetration depth also dramatically dropped from 0.40 mm at 30 m·min^− 1^ to 0.11 mm at 45 and 60 m·min^− 1^. This corresponded to a decrease in Awr and Awp.

**Fig. 10:**
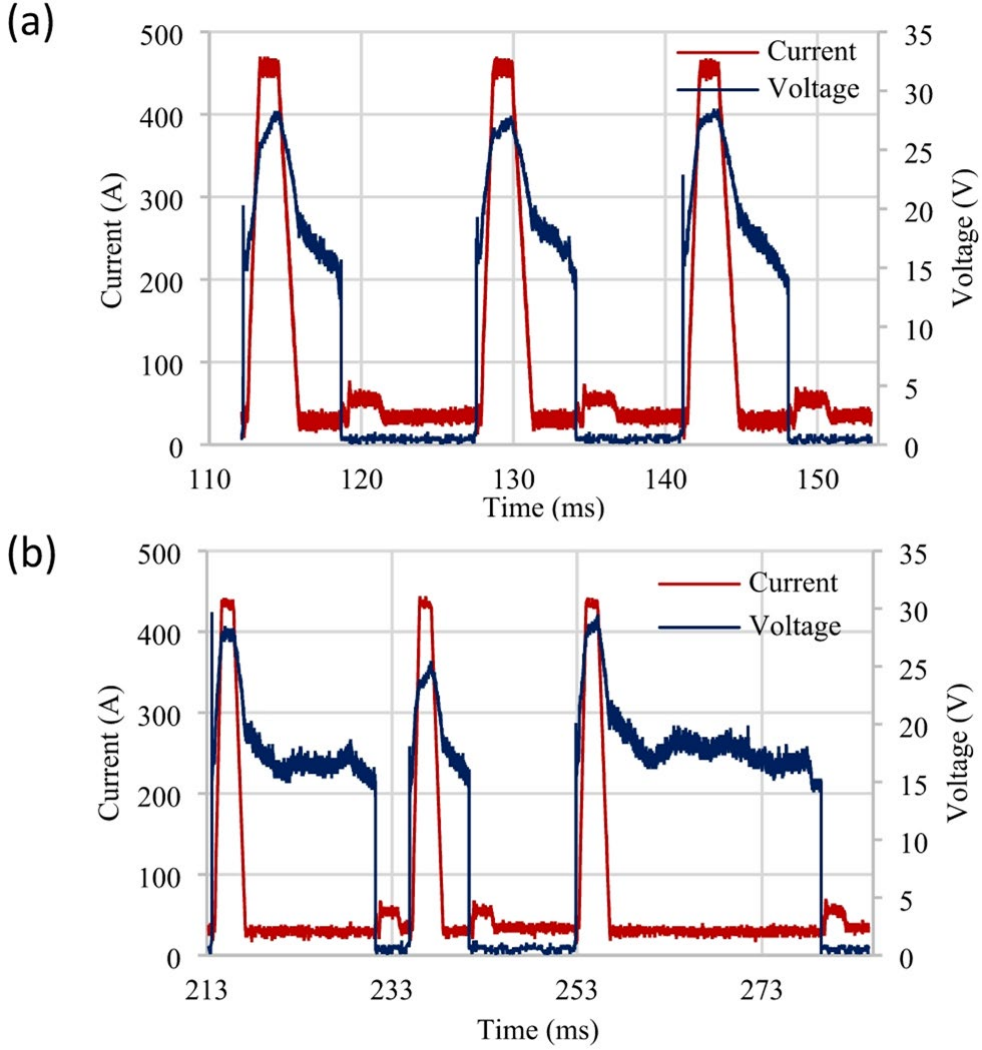
Comparison of CMT characteristic: (**a**) Mean value, (**b**) vd_sc_wait (10 m·min-1).

In addition to the aforementioned impact of electrode speed, the influence of this parameter on the stability of the characteristic profile was also observed, as shown in Fig. 10 (a, b). An unstable characteristic profile Fig. 10 (b) occurred at a 10 m·min^− 1^ electrode speed in the burn phase, which exhibited a significantly longer burn phase duration relative to the SC phase durations. Previous work observed a substantial increase in droplet size and burn phase duration in aluminium alloy welding at 5 m·min^− 1^. This increased surface tension and a deposition geometry composed of multiple continuous spot welds^28,29^.

Analysing characteristics in Fig. 7 (d), burn phase duration decreased with speed until 30 m·min^− 1^, after which it stabilized. Conversely, the SC phase duration increased with speed due to smaller droplet formation and deeper electrode submergence into the weld pool^28^. A cycle length increases

at 10 m·min^− 1^, 20 m·min^− 1^, 45 m·min^− 1^, and 60 m·min^− 1^ was accompanied by a decrease in cycle work. Finally, comparing maximum cycle work with penetration size showed the highest penetration at a speed of 30 m·min^− 1^, with penetration decreasing at speeds of 45 m·min^− 1^ and 60 m·min^− 1^.

#### SC phase current (I_sc2)

The maximum average cycle work Fig. 7 (e), (2065 W) corresponded with the shortest cycle length (13.6 ms), and the minimum average cycle work (1627.2 W) with the most extended cycle length (18.4 ms). This correlation was reflected in the lengths of the burn and SC phases. When the current magnitude of the SC phase was changed Fig. 8 (e) and Fig. 9 (e), deposition height stayed constant (3.6–3.8 mm). Penetration depth increased from 0.38 mm at 20 A to 0.72 mm at 100 A. The contact angle stayed stable between 101° and 104°. The deposition area increased noticeably, from 0.65 mm² at 20 A to 2 mm². Likewise, the Awr deposition area increased from 21.6 mm² to 25.2 mm² at 80 A, with a slight decrease to 24.9 mm².

The I_sc2 parameter controls the current magnitude from when the transferred droplet touches the weld pool until the electrode detaches from the pool. Despite the voltage always reaching minimum (non-zero) values in this phase, an increase in SC phase current led to increased penetration depth. This increase, however, caused subtle changes in deposition height and width.

### Evaluation of parametrical study and WADED

The combination (based on previous findings) of parameters listed in Table 3 was selected to optimize deposition geometry while ensuring the stability of the process. These parameter settings were explicitly chosen to minimize material loss, achieve the desired contact angle range of 90–120°, and prevent significant growth in deposition geometry.

**Table 3:** Table of CMT parameters used for WADED.

I_boost	t_I_boost	I_sc_wait	vd_sc_wait	I_sc2
(A)	(ms)	(A)	(m·min-1)	(A)
430	2.5	35	30	50

**Fig. 11:**
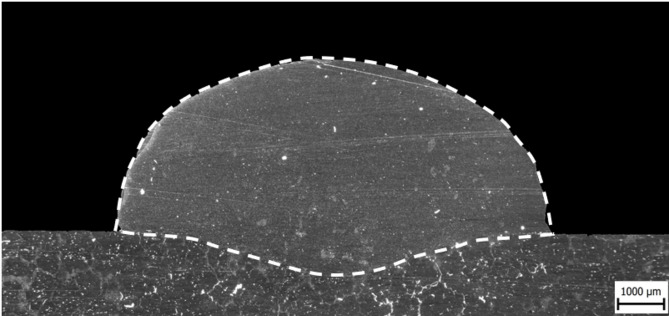
Weld deposited with parameters used for WADED.

**Fig. 12:**
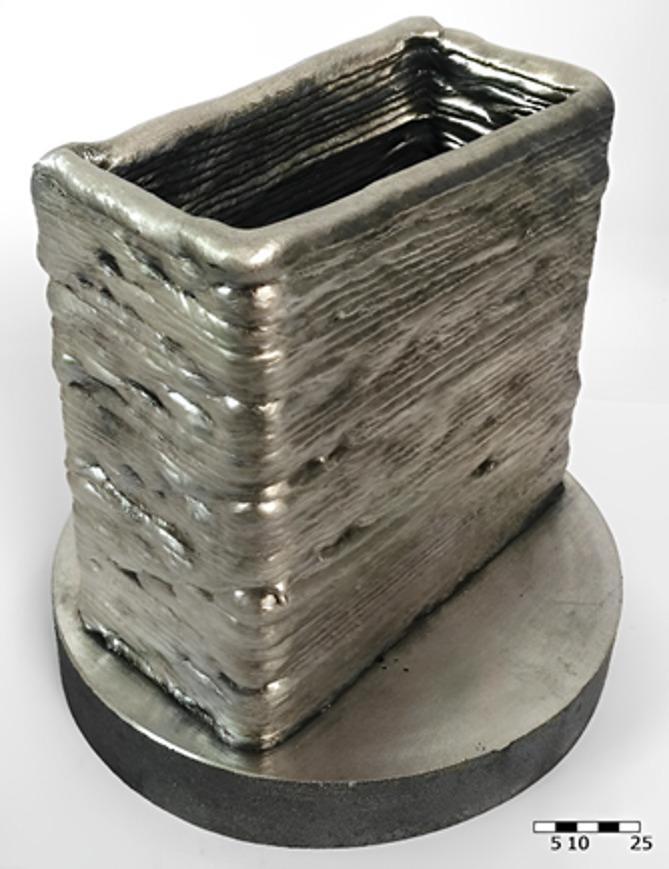
AZ61 thin-walled object fabricated by WADED.

The optimised parameters were implemented, resulting in a stable welding process. WADED benefited from the application of these parameters, leading to improvements in penetration depth, which was evenly distributed across the entire width of the deposition in cross-section, as shown in Fig. 11. The successful deposition exhibited a suitable contact angle of 108°, making it well-suited for thin-walled parts. The final part of Fig. 12 dimensions were 130 × 60 × 130 mm, with a wall width of 9.8 mm, indicating successful prevention of excessive overheating.

However, it is essential to acknowledge that the production process encountered challenges. The presence of impurities in the wire and deviations in the welding trajectory introduced errors during manufacturing. Despite these obstacles, implementing the optimized parameters proved highly effective in achieving the desired deposition characteristics.

### Material analysis

**Fig. 13:**
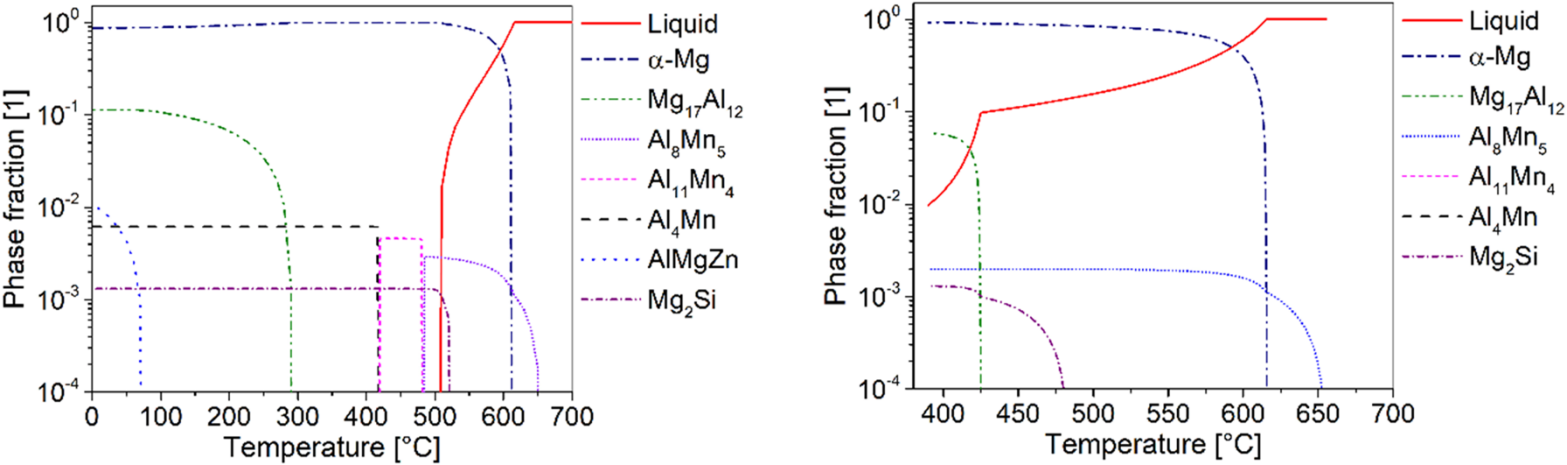
Equilibrium (**a**) and Scheil (**b**) phase calculations for magnesium AZ61 using the average composition given in Table 1 (Fe, Cu, Ni omitted).

Phase fraction calculations for the AZ61 alloy based on the average composition in Table 1 are illustrated in Fig. 13, with (a) representing the equilibrium state and (b) using the Scheil equation. The elements Fe, Cu, and Ni were omitted from the calculations because, although they are common impurities, their content is low.

The calculations indicate that the intermetallic β-phase Mg_17_Al_12_ forms in significant quantities in addition to the Mg solid solution. This phase precipitates directly from the solid solution at temperatures below 300 °C according to the equilibrium calculation. However, at conventional (non-equilibrium) solidification in technical processes, this phase often precipitates primarily from the melt, forming a divorced eutectic β-phase at the grain boundaries^8^. This is supported by the Scheil calculation, where the Mg_17_Al_12_ phase forms at temperatures around 425 °C and, therefore, above the solidus temperature.

Furthermore, the calculations show that Al-Mn phases are formed primarily, with variable stoichiometries (Al_8_Mn_5_, Al_11_Mn_4_ and Al_4_Mn). It is known from practice that mainly Al_8_Mn_5_ is formed, which is stable down to room temperature^30^. With silicon, which typically exists as an impurity in technical Mg-Al alloys, the intermetallic phase Mg_2_Si can form. According to the calculations in Fig. 13, this phase forms primarily from the melt.

The equilibrium calculation also shows the formation of an AlMgZn phase, which precipitates from the solid solution at temperatures below 100 °C. However, this phase does not typically form during conventional processing conditions due to the very low diffusion rate at these temperatures.

**Fig. 14:**
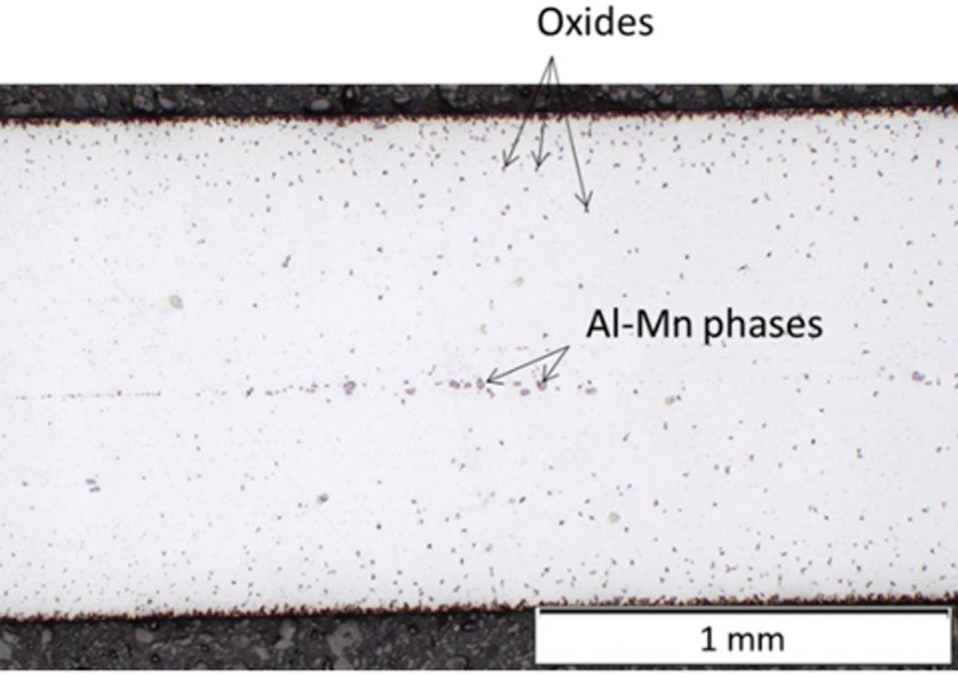
Longitudinal section of the used Mg AZ61 wire.

The longitudinal section of the Mg AZ61 wire, captured using light microscopy, is depicted in Fig. 14. The wire is partially significantly oxidized, with oxides particularly prevalent on the wire surface, decreasing towards the centre. A possible cause could be improper storage of the sensitive Mg wire before processing. Furthermore, band-like arranged blocky Al-Mn phases with a maximum size of 30 μm can be observed, especially in the wire centre. It is assumed that these are primarily formed Al_8_Mn_5_ phases from the casting process, which align along the deformation direction during wire manufacturing. In addition to the larger Al-Mn particles in the wire centre, the same phases can be found finely distributed throughout the wire.

**Fig. 15:**
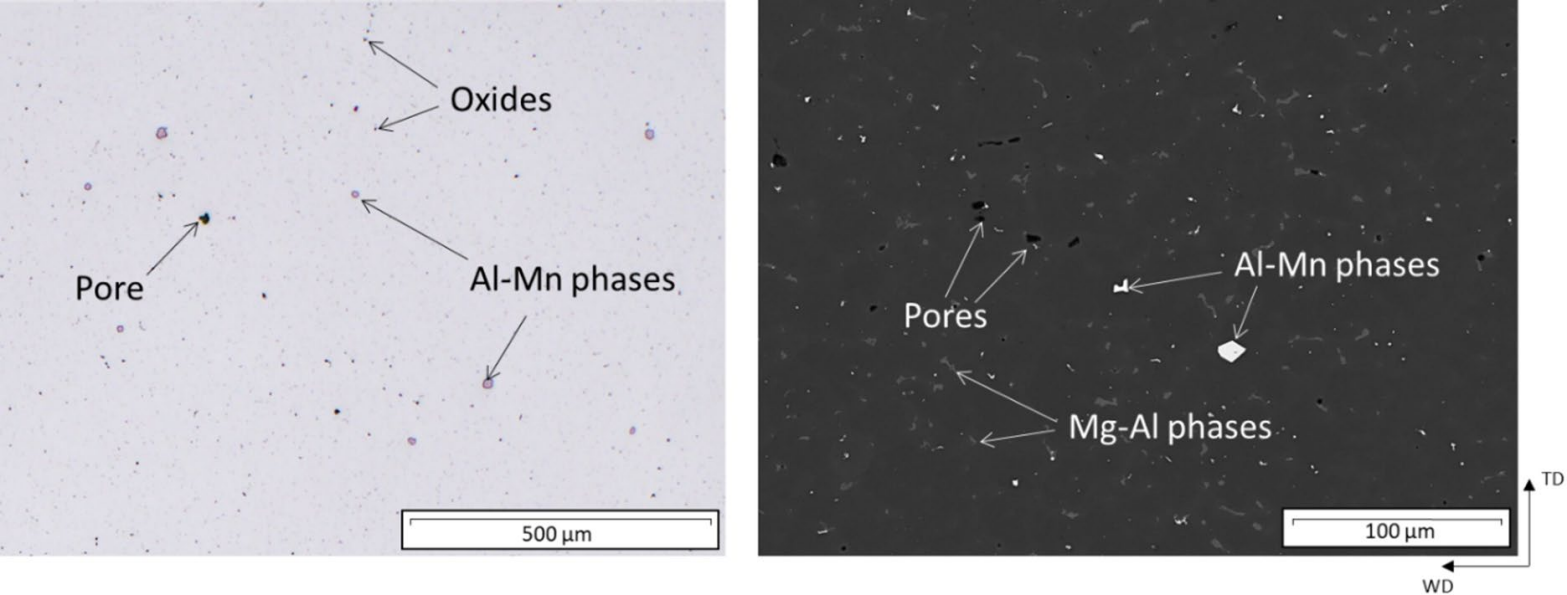
Microstructure of a WADED sample.

Figure 15 shows the microstructure of a WADED sample. The oxides clearly visible in the wires

(see Fig. 14) appear significantly smaller and more homogeneously distributed after deposition. Blocky Al-Mn phases are distributed throughout the material. Due to the high cooling rate of the WADED process, their size is somewhat reduced, reaching a maximum of 20 μm compared with that in the wire. Additionally, Mg-Al phases can be observed at the grain boundaries. It is assumed and supported by the calculations that these are β-phases (Mg_17_Al_12_), which appear as divorced eutectic. The β-phase content is low throughout the sample, so a continuous network does not form, but rather, the phase appears as individual islands. Sporadically, globular-shaped pores with sizes of up to 70 μm and localized shrinkage voids are discernible.

**Fig. 16:**
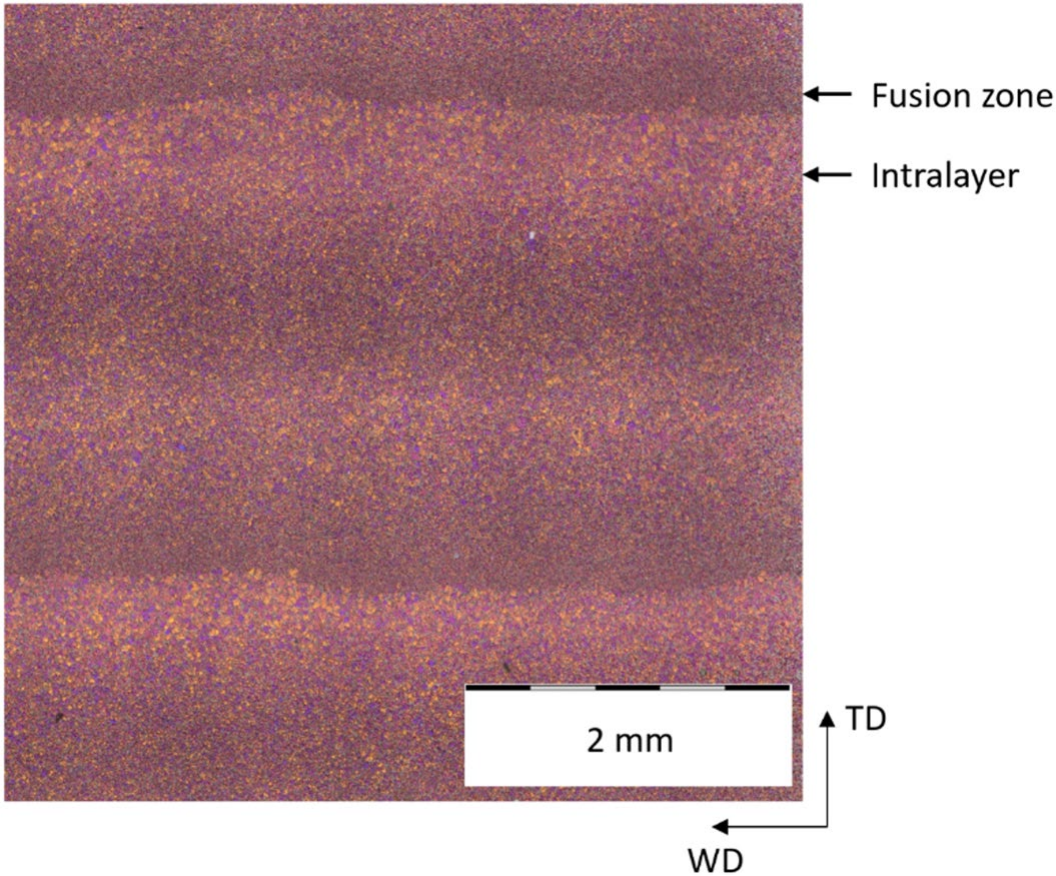
Microstructure of an etched WADED sample observed under polarized light.

The microstructure of an etched WADED sample observed under polarized light is illustrated in Fig. 16. Due to the different grain sizes, the intralayer zone and fusion zone are clearly distinguishable. At the location where a new layer is applied to an already existing layer (fusion zone), the cooling rate is higher, resulting in a fine microstructure in the lower part of the new layer. Farther away from the underlying layer, the solidification rate is lower, resulting in an increased grain size. Similar microstructures were observed for Al alloys processed via WADED by Klein et al.^31^. Graf et al. have found that the zones in WADED AZ91 can also be distinguished by the different proportions

of β-phase^32^. However, this was not investigated in this work. Based on the micrograph, the layer height is approximately ~ 1.5 mm.

### Mechanical properties

**Fig. 17:**
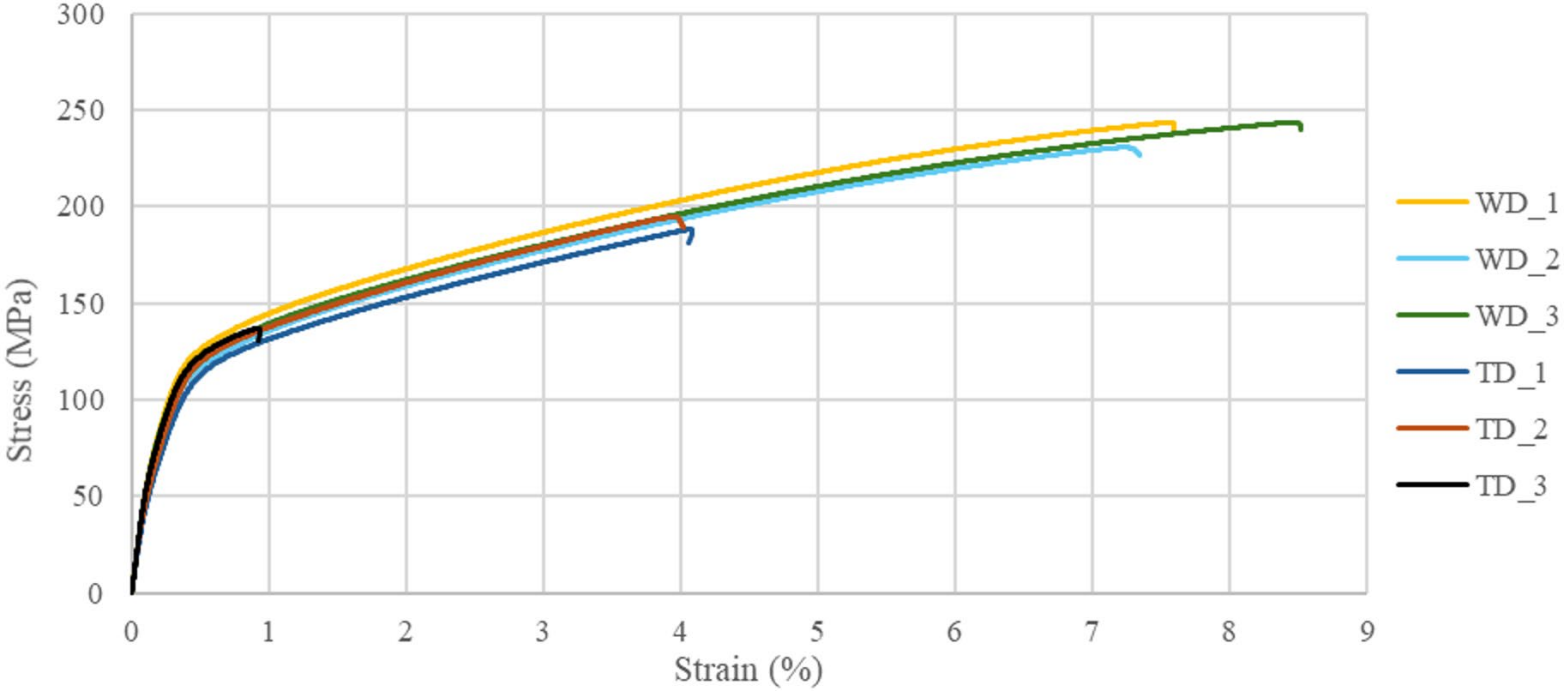
Tensile results of WADED thin-walled object – WD (welding direction), TD (transversal direction).

Three samples oriented in the welding direction (WD) and three in the transversal direction (TD) were tested to obtain the mechanical properties of the AZ61 thin-walled object. The ultimate tensile strength (UTS), yield strength (YS), and elongation (A) were calculated from the stress-strain curves, and the results are listed in Table 4. Figure 17 shows the stress-strain curves of tensile specimens.

The results show that the average yield strength in the WD direction is 114.78 MPa, in the TD direction 110.42 MPa, and a %IPA(YS) anisotropy is 0.139. The average tensile strengths of the material are 239.15 MPa in the WD direction and 173.34 MPa in the TD direction, thus differing more significantly, and the %IPA(UTS) is 0.438. The greatest anisotropy is shown by the elongation %IPA(A) of 0.874, with average elongation values ​​in the WD direction of 7.96% and the TD direction of 4.16%. The average values of yield strength, tensile strength, and elongation for the TD and WD directions, including standard deviations, are shown in the Table 4. Ultimate tensile strength in the TD direction is close to the values ​​achieved in castings. Yield strength in both directions and ultimate tensile strength in the welding direction are higher than comparable casted magnesium alloys but lower than for AZ61 forgings.

**Table 4:** Mechanical properties of AZ61 thin-walled object.

Direction	Ultimate tensile strength UTS (MPa)	Yield strength YS (MPa)	Elongation A (%)
Welding direction (WD)	239.15 ± 7.41	114.78 ± 5.42	7.96 ± 1.79
Transversal direction (TD)	173.34 ± 31.79	110.42 ± 6.14	4.16 ± 2.55

The measured values indicate that the UTS and A of samples made from a thin-walled object exhibit significant anisotropy between the WD and TD directions. However, the overall anisotropy values ​​can be partially distorted by significant differences between the behaviour of the samples in the TD direction.

**Fig. 18:**
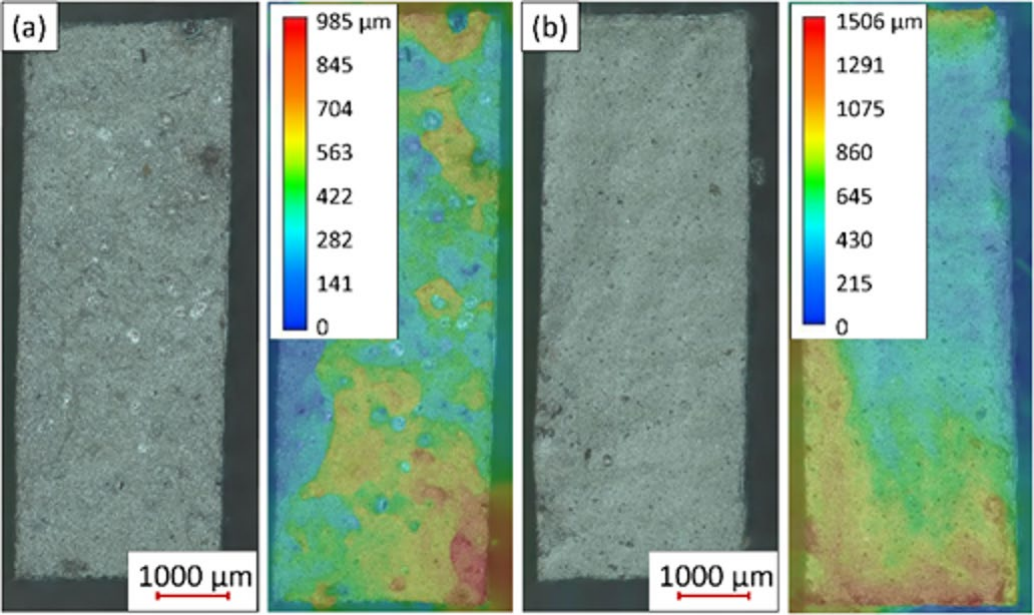
Fracture surfaces after tensile testing (**a**) specimen TD_3 (left: optical image, right: 3D surface profile), (**b**) specimen WD_1 (left: optical image, right: 3D surface profile).

Sample TD_3 shows significantly worse UTS and A values than the other samples in the TD direction. It can be caused, for example, by local defects in the sample. From the fractographic images of the fracture surfaces, as seen in Fig. 18 (a), it is evident that sample TD_3 contains noticeable large local pores both inside the sample and at its edge. Such porous and heterogeneous texture of fracture surface was observed only in sample TD_3. The other samples showed a homogeneous, smooth fracture surface without significant protrusions, similar to sample WD_1, see Fig. 18 (b). Such a defect at the edge of the sample TD_3 can become a stress concentrator and an initiator of crack growth in the material. This local defect would explain the deviation of the UTS and A values ​​of sample TD_3 from the other samples in the TD direction.

However, even if deviated sample TD3 were not considered, the material would still show observable anisotropy in the TD and WD directions. The anisotropy calculated without this sample reaches approximately half the values. The anisotropy values ​​without TD_3 are: %IPA(YS) is 0.139; %IPA(UTS) is 0.227, and %IPA(A) is 0.441. Even such anisotropy values ​​are not satisfactory, but they are not unique. Several authors involved in WAAM production observed a similarly large anisotropy. Yang et al. 26 - Yang observed similar anisotropy values directly on the magnesium alloy AZ31. Yang et al. identified epitaxial columnar dendritic growth along the transversal direction as the leading cause of the anisotropy, whereas mechanical properties showed worse properties in the welding direction. In this work, the mechanical properties showed worse properties in the transverse direction, so another phenomenon probably causes the anisotropy.

Other common causes of anisotropy include the alternation of fusion zone and interlayer zone associated with applying material layer by layer. The material analysis section provides a more detailed description of why layers with different grain sizes were observed in the transverse direction. The anisotropy caused by different grain sizes across the transverse direction is described by Kumar et al., 33 - Kumar who confirmed the connection between grain size and mechanical properties in WAAM samples according to the Hall-Petch relationship. Smaller grains act as barriers to dislocation motion, thereby increasing strength^33 33 - Kumar ^. Conversely, dislocations move more easily in regions with larger grains, leading to a deterioration in mechanical properties.

Another possible cause of anisotropy described by Kumar et al. 33 - Kumar is the effect of residual stresses. A sizeable thermal gradient occurs during manufacturing due to rapid heating and cooling cycles. These thermal cycles induce residual stresses due to uneven contraction of the deposited material.

## Challenges and future work

This article is an initial study on developing suitable parameters for producing thin-walled parts from magnesium alloy AZ61. Based on the experience gained during the experiments, the process will be further optimised to achieve reliable production. The quality of the feeding wire is essential from the point of view of process stability and elimination of local defects. Impurities of any kind in the filler wire are also the cause of unwanted porosity^21^. It is necessary to store the wire correctly to prevent oxidation and clean it before production. For this reason, great emphasis will be placed on quality control of the input wire in subsequent experiments. It is also essential to maintain stable interpass temperatures and consistent conditions throughout production to minimise process instability. Real-time process monitoring further supports this by correlating arc stability with defect formation and helping to identify the sources of instability. Ensuring homogeneous process conditions and applying appropriate heat treatment after production can significantly reduce or completely eliminate anisotropy. Heat treatment reduces residual stresses and homogenizes grain size in the whole part, positively affecting mechanical properties. Guo et al. demonstrated that heat treatment enhanced mechanical properties in AZ80M fabricated using WADED and concluded that an appropriate heat treatment strategy can effectively eliminate anisotropy. Therefore, in follow-up studies, attention will be focused on process control and postprocessing, such as heat treatment or corrosion protection.

Compared to other additive technologies such as Laser Powder Bed Fusion (L-PBF) or Electron Beam Powder Bed Fusion (EB-PBF), WADED has several advantages, including production efficiency, acquisition cost, input material cost and the possibility of producing large-sized parts^35^. Disadvantages include poorer dimensional accuracy, less complexity of the shapes fabricated and poorer surface quality. Mechanical properties of AZ61 alloy produced by L-PBF technology with appropriate heat treatment reach UTS 240 MPa, YS 124 MPa and EL 5.9%^36^. These values are comparable ​​to those achieved by WADED technology if proper heat treatment is applied. The mechanical properties of AZ61 alloy or similar ones produced by conventional technologies are as follows. Forgings of AZ61 alloy reach UTS 295 MPa, YS 180 MPa and EL 12%^8^. Castings are not usually made from AZ61 alloy; the most similar casting alloys are AZ63 and AZ91. The AZ63 sand-cast alloy achieves UTS of 200 MPa, YS of 97 MPa and EL of 6%, and the AZ91 sand-cast alloy achieves UTS of 165 MPa, YS of 97 MPa and EL of 2.5% ^8^. The mechanical properties reported in this paper are higher than those for castings, lower than forgings, and approximately comparable to those produced by L-PBF. It is also worth noting that the optimised parameter set developed in this study was validated only for thin-walled geometries. Its applicability to bulk or thick-walled components, where heat accumulation, residual stresses, and microstructural development differ significantly, has not yet been confirmed. Future work will therefore extend parameter evaluation to volumetric parts and incorporate enhanced thermal-management strategies. Addressing these limitations will be a key step toward establishing a reliable manufacturing chain. Once these issues are resolved, WADED technology, in combination with magnesium alloys, can have a significant industrial impact. Since optimised components in the transportation sector often contain thin-walled and volumetric features, it remains essential to continue improving both the manufacturing process and the associated post-processing routes. In the long term, such developments could minimise material waste and promote sustainability through improved deposition efficiency.

## Conclusion

This paper is focused on developing suitable parameters for producing thin-walled parts from magnesium alloy, identifying the impact of main process parameters on deposition geometry. Magnesium alloy wire AZ61 was deposited on base material by the CMT welding process using various characteristic parameters. The primary conclusions from these results are as follows:

- Boost phase current has significantly affected cycle time and droplet surface tension, resulting in wider and lower deposits. Change in boost duration resulted in rapid material consumption growth, resulting in a bigger deposition area.
- Electrode speed during the boost and burn phase significantly impacted the stability of the welding process and penetration depth. SC phase current directly correlates to penetration depth and has a minor effect on the width, height and contact angle.
- Through optimization of specific welding parameters, a stable welding process was achieved, leading to notable improvements in penetration depth and contact angle suitable for WADED.
- The tensile properties show apparent anisotropic behaviour due to the different grain sizes in interlayer and fusion zones. The average UTS, YS, and A of the welding direction specimens are distinctly superior to those of the transversal direction specimens. The anisotropy of the UTS, YS, and EL for the AZ61 deposit are 0.438, 0.139 and 0.874. Mitigating anisotropy in future applications will require filler wire quality improvement, precise control of thermal conditions during deposition, and implementation of suitable heat treatment strategies.
- Ultimate tensile strength in the TD direction of 173.34 MPa is close to the values ​​achieved in castings. Yield strength in both directions, YS(TD) 110.42 MPa, YS(WD) 114.78 MPa and ultimate tensile strength in the welding direction 239.15 MPa, are higher than those for castings, lower than forgings, and approximately comparable to those produced by L-PBF.

In conclusion, adjusting CMT characteristic parameters can control the heat input and transfer process, thus resulting in the capability to optimize the welding process for WADED application.

## Data Availability

The data that support the findings of this study are openly available in Zenodo Repository at https://doi.org/10.5281/zenodo.15055769. The original version of the authors’ preprint of this study is openly available in the Zenodo Repository https://doi.org/10.5281/zenodo.15311035.
